# Advancements toward a systems level understanding of the human oral microbiome

**DOI:** 10.3389/fcimb.2014.00098

**Published:** 2014-07-29

**Authors:** Jeffrey S. McLean

**Affiliations:** ^1^Department of Microbial and Environmental Genomics, The J Craig Venter InstituteSan Diego, CA, USA; ^2^Department of Periodontics, School of Dentistry, University of WashingtonSeattle, WA, USA

**Keywords:** oral microbiome, metagenomics, metatranscriptomics, stable isotope probing, single cell genomics

## Abstract

Oral microbes represent one of the most well studied microbial communities owing to the fact that they are a fundamental part of human development influencing health and disease, an easily accessible human microbiome, a highly structured and remarkably resilient biofilm as well as a model of bacteria-bacteria and bacteria-host interactions. In the last 80 years since oral plaque was first characterized for its functionally stable physiological properties such as the highly repeatable rapid pH decrease upon carbohydrate addition and subsequent recovery phase, the fundamental approaches to study the oral microbiome have cycled back and forth between community level investigations and characterizing individual model isolates. Since that time, many individual species have been well characterized and the development of the early plaque community, which involves many cell–cell binding interactions, has been carefully described. With high throughput sequencing enabling the enormous diversity of the oral cavity to be realized, a number of new challenges to progress were revealed. The large number of uncultivated oral species, the high interpersonal variability of taxonomic carriage and the possibility of multiple pathways to dysbiosis pose as major hurdles to obtain a systems level understanding from the community to the gene level. It is now possible however to start connecting the insights gained from single species with community wide approaches. This review will discuss some of the recent insights into the oral microbiome at a fundamental level, existing knowledge gaps, as well as challenges that have surfaced and the approaches to address them.

## Introduction

**Figure 1:**
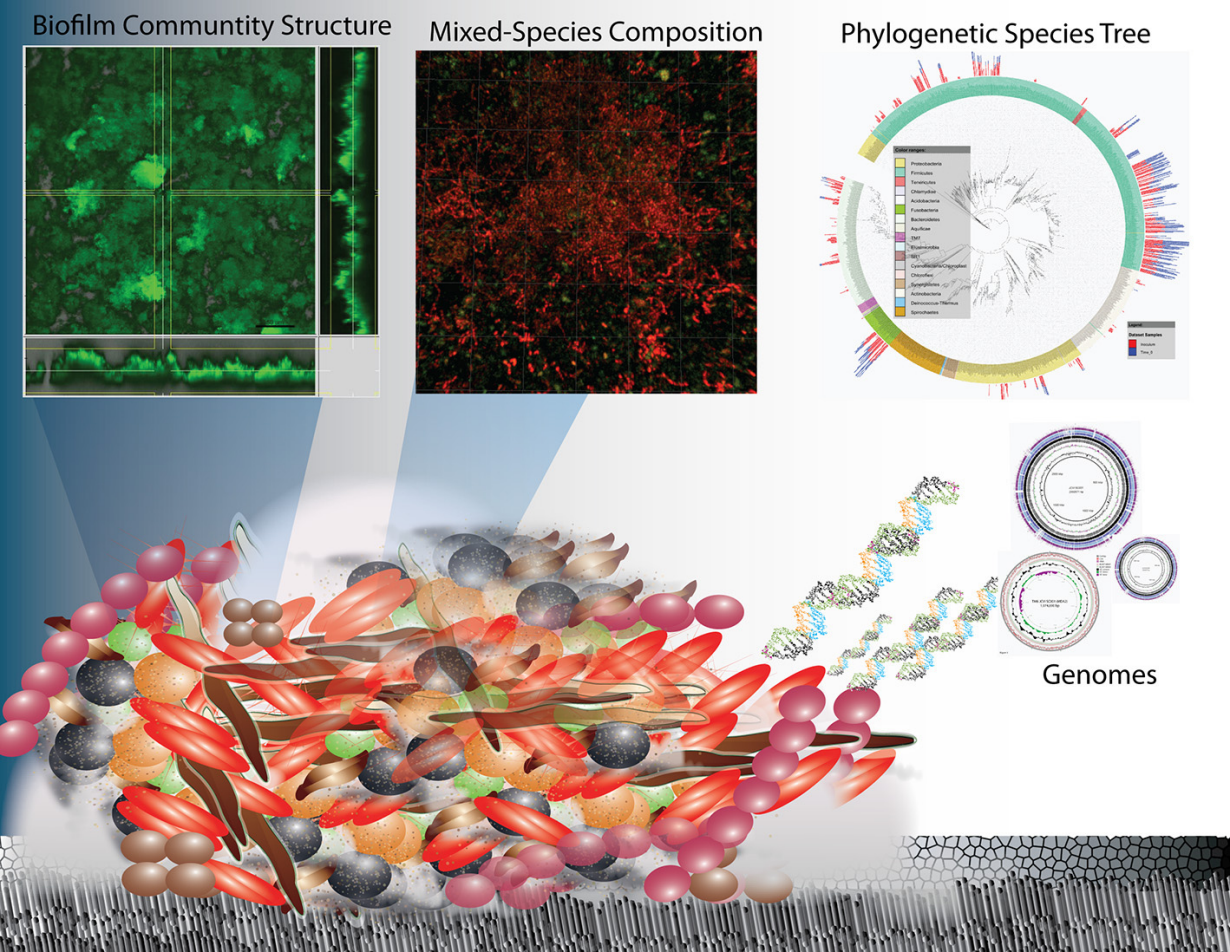
**Overview schematic representation of the structure and complexity of oral biofilms on enamel surfaces in terms of physical heterogeneity and species diversity**. Research on oral biofilms has encompassed biofilm structure and function at the macro-scale to the species diversity through 16 rRNA sequencing and their genetic potential (whole genome sequencing and metagenomics) and is now progressing to the functional activity at the molecular level (metatranscriptomics).

Oral biofilm communities constitute dynamic, multiple-species metabolic networks with a multitude of interconnected functions (Figure 1). The species types, abundance, and activities of microbes are a function of the environmental (physical, chemical, and biological) parameters, including the carbon and other nutrient resources available. Oral communities may exhibit large and rapid changes in composition and activity both temporally and spatially and are developmentally dynamic with the human host (Xu et al., 2014). These complex, non-equilibrium dynamics are consequences of several factors, including the temporal frequency of host and diet, the rapid response to changes in pH, bacteria-bacteria interactions and on a larger time frame, genetic mutations and horizontal gene transfer that confer new properties to strains. Understanding the combination of host and environmental factors that drive the overall balance of biofilm communities represents one of the grand challenges in microbial ecology. Unfortunately, most of what is known about biofilm function has been extrapolated from mono-species culture studies. This is largely because the methods available for biofilm analysis have lacked the sensitivity and/or resolution required to unravel the complexity of these mixed species biofilms. In fact, until recently, the approaches being used lacked sufficient ability to capture the behavior of even known species within a background of a mixed community.

Biofilm structure, function and microbial species composition are all areas that have been and continue to be addressed, with the goal of enabling an understanding of the species interactions that ultimately govern biofilm communities. This is information that is critical to enhancing beneficial biofilms as well as combatting harmful ones. Recent development of advanced methods to be discussed here, in part has allowed us to begin to advance the study of multiple species biofilms, and begin to ask “who is there?”, “who is active?” and “what processes are active?” In addition to having significant health related relevance, dental plaque is one of the best-described microbial communities (Marsh and Bradshaw, 1995; Kolenbrander, 2000; Kolenbrander et al., 2007; Kuramitsu et al., 2007; He and Shi, 2009; Palmer, 2009, 2014; Kuboniwa et al., 2012) and the breadth of fundamental research cannot be covered in this review. It is clear that oral plaque presents a very convenient system for demonstrating the species interactions and functional analysis of multi-species communities in general (Foster et al., 2003; Kolenbrander et al., 2007). For example, an oral biofilm on enamel for example is a much better characterized system than a natural community in marine or soil influencing other mineral surfaces in terms of: (1) the biochemical reactions leading to dissolution; (2) the identity of species present; (3) the number of genomes available; and (4) the ease of conducting biological experiments.

**Figure 2:**
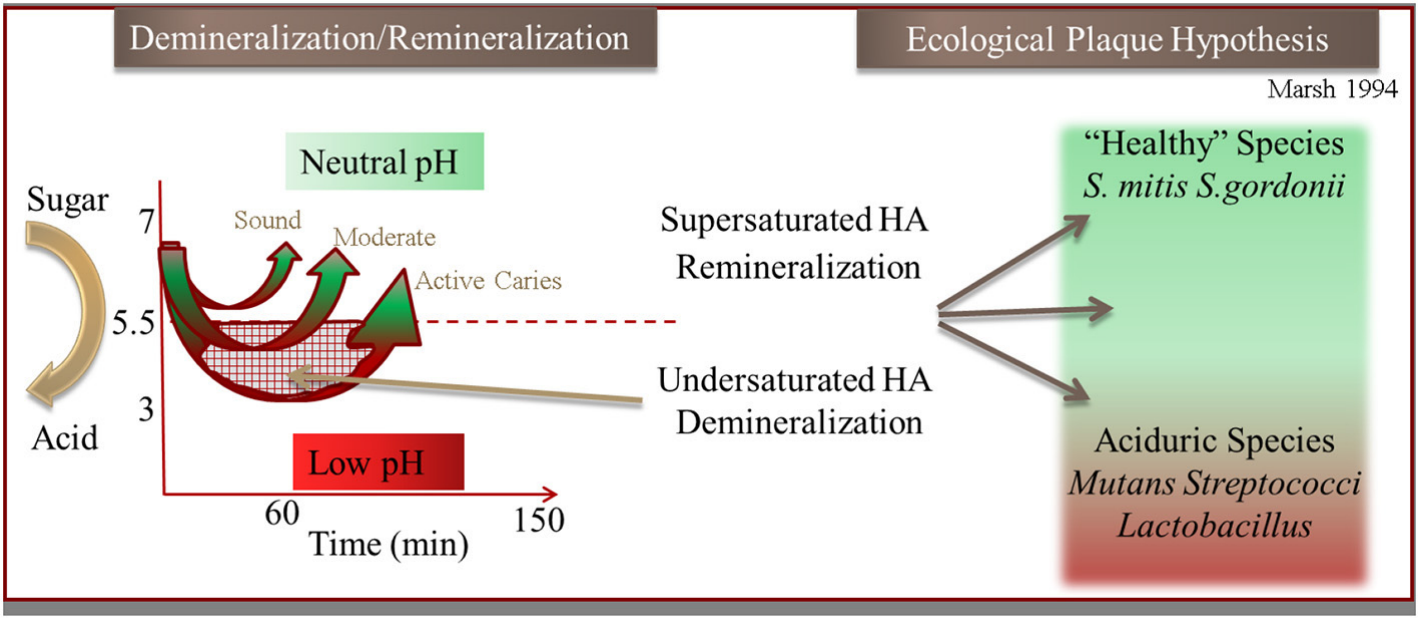
**Illustration of the hydroxyapaptite (HA) demineralization and remineralization process that occurs through the pH cycling (“Stephan Curve”) in relation to the Ecological Plaque Hypothesis (adapted from Marsh, 1994)**.

Processes in the oral cavity related to human disease are predominately driven by reactions occurring within complex microbial biofilm communities in contrast to a few diseases that are the result of a single species. Most studies have been predominately conducted on model systems containing single species which greatly advance our understanding. Bacteria within oral biofilms have been described as “good” (i.e., protective) common components of healthy microbiomes (He et al., 2009, 2010, 2014), and also “bad” as in the case of *Streptococcus mutans* with many falling between these designations. Dental caries is generally now considered a polymicrobial disease that arises when there is dysbiosis and the communities shift metabolism in harmful ways. Under such conditions, the antagonistic biofilms often display enhanced resistance to antibiotics, and as such become the etiological agents of many serious human diseases, including cystic fibrosis, periodontitis, otitis media (inner ear infections), and bacterial endocarditis, to name a few. Tooth decay (dental caries), which is the loss of enamel that is composed of the mineral hydroxyapatite (HAP), is one such polymicrobial mediated process that is thought to be caused by a shift in biofilm populations from “good” to “bad.” The shift to more acidogenic (acid generating) and aciduric (acid tolerant) species is thought to drive demineralization of the HAP crystals through an increase of acidic end products of fermentation. The physical and ecological model (Marsh, 1994) of this process is described briefly in Figure 2. This reproducible pH response observed after a sugar rinse has been acknowledged for nearly 80 years and is defined as the Stephan Curve (Stephan and Miller, 1943). This rapid cycling of pH has been well documented both *in vivo* and within *in vitro* collected plaque. Early research shows that this cycling is present within individual species to varying degrees (Kleinberg, 2002). Having defined a physiological and ecological model of supragingival plaque in relation to enamel demineralization has driven testing of these hypotheses. Despite many years of research however, demineralization of enamel remains enigmatic in terms of governing critical activities that occur within the diverse community.

The study of individual oral species and now entire communities in the oral cavity has benefited greatly from recent approaches based in genomics and bioinformatics. Many hurdles still remain however and overcoming these will rely on technological and experimental advances that disentangle the immense complexity of a multispecies biofilm. This review mainly covers the supragingival bacteria involved with dental caries and the resultant demineralization of enamel which, due to the reasons mentioned above, is most well described. Many of the challenges are also applicable to periodontal disease and other diseases related to the human microbiome. Understanding the role of bacteria in caries is inherently and extensively interdisciplinary, involving microbiology, molecular biology, genomics, proteomics, and metagenomics, as well as microbial physiology and biochemistry. This work has involved the development and use of methods for the study of the structure and function of these complex biofilms on one hand, and for the physiology and genomic characterization of individual community members on the other. Both top-down and bottom up approaches are clearly needed to more fully understand the abiotic and biotic factors that contribute to the fundamentally important process of tooth decay.

## Current knowledge of bacterial metabolic processes leading to demineralization

It is clear that demineralization exhibits a strong correlation with biofilm induced pH reduction, but in reality, a detailed understanding of the metabolic processes responsible for pH cycling (Figure 2) is still lacking. Most of what is known about bacterially mediated demineralization in the oral cavity comes from the research on a few model organisms. It is commonly reported that lactic acid is the organic acid produced by the community and that lactic acid accumulation at the biofilm-enamel interface is responsible for the large pH shift leading to enamel demineralization. This is a very simplified view since only a few studies have addressed which organic acids are produced during the metabolism of the common sugars (glucose and/or sucrose) under conditions found in a biofilm. None have been able to quantify the absolute concentration of multiple organic acids in live biofilms, especially non-invasively in a temporally or spatially-resolved manner. The metabolite profiles of a supra-gingival oral community *in vivo* under “diseased” (low pH) and “healthy” (neutral pH) environmental conditions are therefore unknown and represent a challenge. Furthermore, we know that there is a large diversity of species with varying metabolic capacities encoded in their genomes that could modulate pH.

A number of studies support the contribution of other organic acids toward demineralization even in the model species *S. mutans*. It has been shown early on that *S. mutans* given excess glucose exclusively produced lactic acid under aerobic conditions (Yamada and Carlsson, 1975; Yamada et al., 1985). Under anaerobic conditions lactate, formate, acetate, and ethanol are formed, with lactate making up less than 50% of the total acids produced. Since biofilms are typically stratified with respect to oxygen concentration, these findings suggest that the outermost biofilm layer (where oxygen concentrations are higher) would possibly present high levels of lactic acid metabolites while internal regions of the biofilm that are closer to the enamel surface and exposed to reduced oxygen tensions would more likely give rise to other acids in combination with lactate. Furthermore, overall higher yields of acid occurred when cells were grown anaerobically rather than aerobically with acetate and formate being the dominant acids present (Yamada and Carlsson, 1975; Yamada et al., 1985). An NMR study of perchlorate extracts of oral biofilms demonstrated the utility of ^1^H-NMR for the simultaneous analysis of ~30 chemical constituents, including lactate and other corrosive organic acids (Silwood et al., 1999). Their results indicated that lactate, acetate, pyruvate, propionate, formate, and *n*-butyrate are produced in abundance, with acetate and formate being produced at higher concentrations than lactate. Considering the larger dissociation constants of these organic acids, they concluded that formic and pyruvic acids contribute significantly to the decreased pH values. The authors of this NMR study stated that previous studies of carious lesions have failed to detect and therefore consider the contribution of formic and pyruvic acids to demineralization of tooth surfaces (Silwood et al., 1999). While supporting the hypothesis that acids other than lactic acid are likely to be important players in cavity formation, this approach could not differentiate between acids produced extracellularly where they can interact with surfaces from those retained within the cytoplasm of the cells. This information is essential for determining which acids are most important in demineralization processes.

Recently, our laboratory has conducted experiments to gain insight into the spatial and temporal dynamics of metabolism. Novel NMR based metabolite measurements of sugar metabolism were performed in a non-invasive, real-time manner on active oral biofilm communities including, a *S. mutans* biofilm model with temporal and spatial resolution (McLean et al., 2008) as well as plaque samples derived from healthy children (McLean et al., 2012). These types of non-invasive measurement approaches combined with other destructive measures are essential to gain an understanding of the metabolic processes that are responsible for cavity formation. Further efforts to obtain the full suite of untargeted metabolite profiles temporally and spatially will help reveal the pathways and processes active at low pH and also during the rapid pH recovery phase which is important for a “healthy” functioning community.

## Dynamic metabolic and population shifts leading to sustained demineralization and the ecological plaque hypothesis

Possibly the best example in the development of overarching hypotheses governing the shift in a microbial population from a healthy (balanced neutral pH) to a “diseased” state (extended periods of low pH) comes from extensive studies of the oral cavity, beginning with the specific plaque hypothesis developed by Loesche (1976, 1979). This hypothesis implicated that only a few species were responsible for caries. *Streptococcus mutans*, identified very early as a major player in the onset of caries, has been the primary subject of most caries studies. With further investigations it became evident that many other bacteria termed “cariogenic or oral pathogens” such as *Streptococcus sobrinus* and *Lactobacillus* spp. exhibit low pH metabolic behavior similar to that of *S. mutans*. The non-specific plaque hypothesis developed years later (Theilade, 1986), implicated the microbial community as a whole as being responsible for caries. Marsh and colleagues later developed the “ecological plaque hypothesis” (Marsh, 1994) stating in essence that oral caries and periodontal diseases arise as a result of environmental perturbations that lead to a shift in the balance of the resident microflora. Key features of this hypothesis are that (a) the selection of “pathogenic” bacteria is directly coupled to changes in the environment; and (b) diseases need not have a specific etiology, any species with relevant traits can contribute to the disease process. Thus, the significance to disease of newly discovered species can be predicted on the basis of their physiological characteristics. For caries, the environmental perturbation arises from the intermittent introduction of dietary sugars during feeding leading to cycling of pH (Stephan, 1945). If the pH remains low for sustained periods, a shift in the bacterial populations to more aciduric organism is thought to occur (Marsh and Bradshaw, 1997; Kleinberg, 2002). This was documented to some extent through laboratory culturing studies in chemostats using defined mixed communities. It is envisioned that under disease conditions *in vivo*, the low pH would drive the dissolution of calcium and phosphate in the hydroxyapatite crystalline structure of the tooth and ultimately lead to cavitation. Cariogenic bacteria are then thought to thrive under these acidic conditions, increasing in proportion and worsening the diseased state. This has proven extremely difficult to validate *in vivo*.

From numerous 16S rRNA gene profiling and clinical investigations it is clear however that certain acidogenic and aciduric species such as *S. mutans* and *Lactobacillus* spp. are highly correlated with active caries. There are however, many other species that are likely to be relevant as evidenced by the diverse microbial populations present in caries in young children that include *Actinomyces, Fusobacterium, Porphyromonas, Selenomonas, Bacteriodetes*, and *Haemophilus* (Corby et al., 2005). Through Denaturing Gradient Gel Electrophoresis (DGGE) profiling at least 30 species were found in active caries sites including *Gemella, Kingella, Leptotrichia, Streptococcus*, and *Veillonella* (Li et al., 2007). More recent high throughput sequencing studies are further supporting the diversity of bacteria that may be involved with caries through association studies (Tanner et al., 2011; Gross et al., 2012; McLean et al., 2012). Clearly, substantial evidence is available to support the hypothesis that cariogenic activity of an oral biofilm could be impacted by multiple members of the community.

Overall, the contributions of each species to the healthy and diseased state still remain largely unknown. For example, while the properties of many of the identified cariogenic bacteria such as *S. mutans* are known in pure culture (Loesche, 1986) as well as differences in strains (De Soet et al., 2000), knowledge of their physiological and metabolic behaviors in a diverse multi-species dental biofilm is scarce. It is clear from investigation in mono- and dual-species model systems that *in-vivo* characteristics can be greatly impacted by other members of the community. Commensal bacteria in dental plaque biofilms may impact the processes of acid tolerant species (cariogenic pathogens) (Takahashi and Nyvad, 2008). This can be accomplished indirectly by modulating the activity of cariogenic species as well as impact the pH drop by the production of alkaline byproducts such as ammonia from arginine (Takahashi, 2003). It has also been shown that it is possible to impact virulence in a more direct manner such as the example of *Streptococcus sanguinis* inhibiting *S. mutans* growth through the production of H_2_O_2_(Kreth et al., 2005). Linking the functions observed in mono-species cultures to their activities within a population is a challenge that is now technically possible with advances in sequencing and bioinformatics there are still fundamental gaps yet to be addressed which is the subject of the following sections.

## Current needs to address the polymicrobial problem

The oral microbial system is an ideal system to study the driving forces influencing homeostasis and dybiosis. In order to gain knowledge to support or refute the hypothesized mechanisms behind the ecological plaque hypothesis for example, one needs to know all the players and be able to track their behavior. Since most microbes remain uncultivated, little is known about these species except for their 16S rDNA sequence. Furthermore, although many model bacteria that are known to be one of the species associated with a particular condition *in vivo*, these bacteria have only typically been characterized in the laboratory as pure cultures. Validating whether these observed laboratory characteristics are actually maintained *in vivo* in the presence of a mixed microbial community is a challenge and this is where the techniques have been lacking. Specifically, major outstanding questions in the study of mixed microbial communities include:

1. What is the behavior (metabolism) and gene expression of known model species when they are active within a mixed species microbial community?
2. What is the role of currently uncultivated organisms and their contribution to the overall function of the community?
3. How does a stable microbial community shift to an undesirable state? (which species, metabolic pathways, and genes are involved?) Can this species shift be predicted?

But why has it been so difficult to move from the study of single-species to the study of natural, mixed-species communities? In addition to the issue of unknown (uncultivated) taxa, there are the simple physical problems related to the size of microbes and microbial communities. Biofilm communities are often only a few to 100 μm in height and are composed of individual members at the micrometer size range that are in close contact with one another. Thus, the challenges in sensitivity and resolution are great, and the development of appropriate tools for the study of biofilms in a minimally invasive manner has moved slowly. Furthermore, while the questions that need to be answered are easy to phrase, the pathway to answering them is not easy. There are a few major scientific challenges that must be met before it will be possible to move confidently from the study of single to multi-species biofilm studies which are briefly stated here and covered in more detail in the following sections:

1. **The need for microbial genomes as references for community based studies**. In order to more fully grasp microbial taxonomic and gene diversity as well as to provide a means to assign metagenomic (DNA) and metatranscriptomic (mRNA) reads to a given species, reference genomes are critical. The rapid growth of metagenomic and metatranscriptomic sequencing has revealed a need for more reference genomes in order to be able to assign reads to genes and/or bacterial species and therefore identify possible taxon-related function within a given biofilm. As reference genomes continue to grow as they have in the last 5–10 years with advanced approaches, the complete inventory of genes and their linkage with a particular species/strain within a given microbiome may ultimately be possible to determine.
2. **The need for tools to address the large fraction of uncultivated species**. While many new microbes can be identified through 16S rDNA gene based diversity analyses, further research on many of these microorganisms is hampered by an inability to uncover their culture requirements. In the absence of a culture, physiological inferences can be made through the genome of an uncultivated species. Further advances on culture methods as well as methods such as single cell genomics capture a representative genome are needed to make substantial headway in this area given the vast amount of known uncultivated diversity present.
3. **The need for mixed-culture laboratory model biofilm communities**. Understanding individual species function, metabolism and gene expression profiles in a biofilm is a necessary step in the study of polymicrobial processes. Importantly, multi-species models that are reproducible and stable will allow for hypothesis testing and functional validation of observations made *in vivo*. Models that contain uncultivated phylotypes (those species only known by their 16S rRNA gene sequence) are indeed more comprehensive and valuable. Once again, it should be noted that the need for cultivated strains and reference genomes from these model systems is key in order to more fully understand the dynamics within laboratory models and the role uncultivated species play.
4. **The need for methods that enable species level resolution of function within biofilm communities**. Overall, it is likely that model species modify their behavior in the presence of other community members. How (and how much) the behavior of a given microbe changes in the presence of the other members of a complex biofilm community is not presently known. This knowledge gap is a result of the inability to track the behavior of many individual species. There are few approaches available to understand the behavior of cultured isolated organisms when they are put back in a complex community. A number of exciting new approaches predominately based on deep sequencing technologies have allowed us to ask questions about “who” is there and “what” are they doing within a diverse community. The ability to monitor biological functions and link this observed activity to the identity of the species responsible is an area that is just starting to become available through techniques such as nucleic acid base Stable Isotope Probing (SIP) and more recently metatranscriptomics.

## Challenges to progress on community level microbiology: the uncultivated and unknown majority

With the increasing advances in DNA sequencing technologies, combined with the reduction of sequencing costs, access to the microbial world has greatly expanded to reveal an unprecedented microbial diversity across nearly every environment. Pioneering large scale environmental shotgun sequencing with the Sargasso Sea pilot study (Venter et al., 2004) and the larger Global Ocean Sampling (GOS) expedition (Rusch et al., 2007), focused on marine surface waters. Recently the Human Microbiome Project (HMP) (Turnbaugh et al., 2007; Human Microbiome Jumpstart Reference Strains et al., 2010; Consortium, 2012a,b) efforts have revealed remarkable microbial diversity within and on the human body. Initial metagenomic HMP efforts also made it painfully obvious that there were major gaps in terms of the number of available reference genomes. Reference genomes for the oral cavity are critical for capturing species diversity, gene content (metagenomics), gene expression profiles (metatranscriptomics), expressed proteins (metaproteomics) and small molecules (meta-metabolomics). Without annotated genes from reference genomes to assign reads and proteins to, there is no taxonomic information obtained. These are referred to as “orphan reads” and currently a large proportion of sequences from microbial community studies fall within that category.

Depending on the environment being studied, only a small percentage of the microbes visible under the microscope are likely to be easily domesticated in the lab. It has been dubbed the “great uncultivated majority” (Whitman et al., 1998), “dark matter of life,” and “microbial dark matter,” which includes microbes and even entire divisions of bacterial phyla that have evaded cultivation. Many candidate phyla for example have yet to have a single representative whole genome sequence. Since the realization of this missing diversity in culture attempts (Staley and Konopka, 1985), estimates now indicate only 1–10% of known bacterial species (Rappe and Giovannoni, 2003) are thought to be currently cultivated. Fortunately, great progress is being made for some bacterial communities; for example, roughly half of bacterial species within the human oral cavity have been cultivated (Dewhirst et al., 2010). Since this vast majority of bacteria in the environment as well as those associated with the human microbiome have eluded standard culturing approaches, their physiology and their gene content are unknown. In the absence of culture-based physiological analyses, the functional roles of these uncultivated species remain mysterious despite their apparent correlations with important processes. These problems have become the limiting step in studying ecology-based community activities. In the “best of all worlds,” reference genomes would be obtained, then used to link function to phylotype for uncultivated microbes. Eventually the information could be used to guide successful cultivation of these abundant or rare uncharacterized microbes.

## Current knowledge on the species in oral biofilms

Through isolation/culture and culture-independent methods, the species present as attached cells in biofilms within the oral cavity have been estimated to comprise a diverse community of more than 700 phylotypes inclusive of bacterial and archaeal domains, although less than 100 phylotypes are found in a typical individual (Dewhirst et al., 2008). The most comprehensive database for 16S and genomic data for the oral cavity is the Human Oral Microbiome Database (HOMD; www.HOMD.org) (Dewhirst et al., 2008). To date, from this curated database, there are 691 total taxa (98.5% similarity in 16S rRNA), 344 named taxa, 112 cultivated but unnamed taxa, and 232 uncultivated taxa (phylotypes). The oral cavity is one of the most well covered microbiomes to date with a total of 392 taxa that have at least one reference genome with the total genomes across the oral cavity approaching 1500 (Human Microbiome Jumpstart Reference Strains et al., 2010). The HMP initiative (http://commonfund.nih.gov/hmp/index) leveraged the recent advances in genomics to allow for a far more comprehensive survey of the microbial species and their associated genes present in the oral cavity both through the generation of hundreds of new reference genomes from cultivated oral strains to deep metagenomic sequencing of human subjects(Consortium, 2012b). Many databases cover the extensive data produced from nine distinct sites in the oral cavity from the roughly 230 healthy western volunteers to serve as a baseline for a “healthy” human microbiome. The 16S rRNA and shotgun datasets as well as assemblies are available on a number of public databases which house comparative metagenomic tools. These include the HMP Data Analysis and Coordination Center (http://www.hmpdacc.org/), the Integrated Microbial Genomes Human Microbiome Project (https://img.jgi.doe.gov/cgi-bin/imgm_hmp/main.cgi), the JCVI METAREP (http://www.jcvi.org/hmp-metarep/) to name a few.

## Single cell genomic sequencing: capturing reference genomes of rare and uncultivated microbes

Given the number and diversity of taxa found within biofilms, it becomes important to know the members of these communities (and their activities) at a higher level of resolution than allowed by the most commonly used detection and identification methodologies. Determining what species and what genes are present are some of the initial strategies. Culture-independent surveys using the 16S rRNA gene as a marker are currently the most widely used approach however genetic strain differences reflecting potential different metabolisms and phenotypes are often difficult to resolve due to this gene being highly conserved amongst many bacterial strains. Quantitative PCR and direct culturing are focused on either a handful of predetermined species or what can be readily cultivated which we already know to be only a minor portion of the species in any given environment. Metagenomic is limited with regard to accurately predicting taxonomic affiliation at the species or strain level from highly diverse and complex datasets with short read sequencing technology. In addition, whole genome comparative genomic studies on the evolution and transmission of a low abundance organism of interest that resides in a microbial community requires substantial amounts of DNA or a cultured strain from the community which often cannot be obtained.

Sequencing from single bacterial cells, first achieved in 2005 (Raghunathan et al., 2005). This breakthrough was enabled by the development of the MDA reaction (Dean et al., 2001, 2002), which can amplify a single genome copy more than a billion fold enabling sequencing of DNA from very low (femtogram) levels (about the amount of DNA in a single bacterial cell). Bacteria that have not been cultivated by conventional culturing techniques are currently the central target of single-cell genomics (Lasken et al., 2005; Raghunathan et al., 2005; Hutchison and Venter, 2006; Ishoey et al., 2008; Lasken, 2012) The recent advancements in DNA sequencing of single bacterial cells has accelerated the study of uncultivated microbes (Lasken, 2012), providing genomic assemblies for species previously known only from 16S rRNA clone libraries and metagenomic data (Marcy et al., 2007; Podar et al., 2007; Binga et al., 2008; Eloe et al., 2011; Youssef et al., 2011; Dupont et al., 2012; McLean et al., 2013a; Nurk et al., 2013b; Rinke et al., 2013). Using these approaches, the so-called “dark matter of life” which represents uncultivated microbes and even entire divisions of bacterial phyla (candidate divisions and candidate phyla) are slowly being revealed with assembled genomes. The single cell sequencing approach has had a number of notable successes allowing full and partial recovery of genomes from many elusive Candidate bacterial groups at the phylum level including but not limited to; oral TM7 (Marcy et al., 2007), oral SR1(Campbell et al., 2013), and TM6 (McLean et al., 2013a) from a drinking water distribution system.

**Figure 3:**
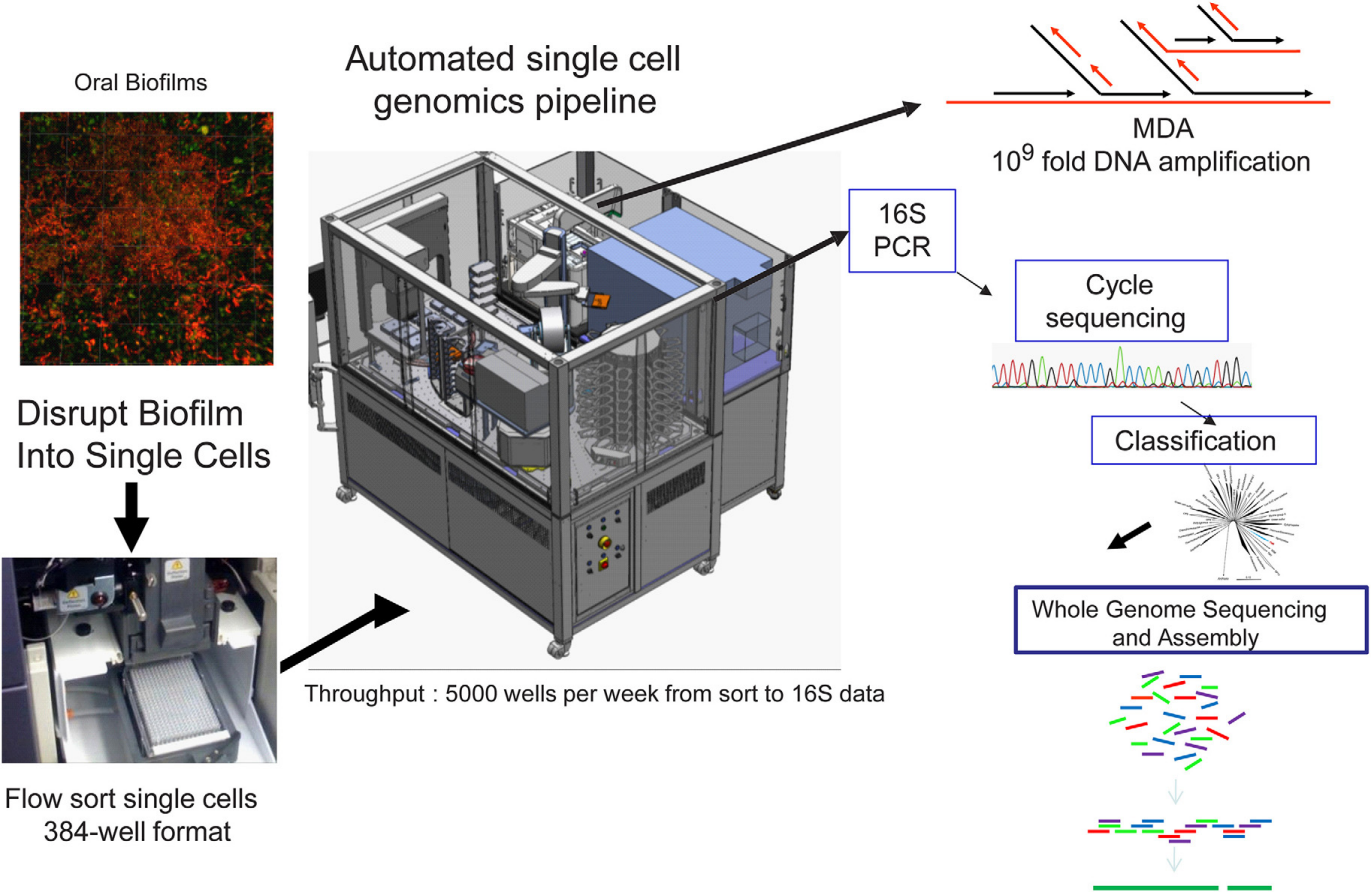
**Workflow of the high-throughput Single Cell Genomics Platform at the J. Craig Venter Institute**. Custom integrated Agilent Technologies BioCel 1200 liquid handling automated platform for high throughput single cell genomics. The BioCel platform allows processing of more than 5000 single cells per week through a multi-stage protocol that includes multiple displacement amplification (MDA) of DNA, MDA dilution and 16S rDNA PCR. After classification of 16S rRNA gene sequences, candidate genomes can be deeply sequenced followed by assembly and annotation of generated contigs. Modified from McLean et al. (2013b).

A new high throughput and highly automated platform was recently reported for sequencing and assembly of single cell genomes of bacteria (McLean et al., 2013a) and viruses (Allen et al., 2011) (Figure 3). The workflow consists of: (1) delivery of single bacterial cells (single cell genomics) or small pools of cells (mini-metagenomics) (McLean et al., 2013a) into 384 well microtiter plates by Fluorescence Activated Cell Sorting (FACS); (2) use of a robotic platform to perform 384 well automated cell lysis and amplification of DNA by the (MDA) method (Dean et al., 2001, 2002; Hosono et al., 2003) to create libraries of genomic DNA derived from single cells; (3) PCR and cycle sequencing of 16S rRNA genes to profile the taxonomy and diversity of the libraries; (4) selection of candidate amplified genomes for whole genome sequencing; and (5) sequencing and assembly of selected genomes using assembly tools designed specifically for MDA amplified single cells (Chitsaz et al., 2011; Bankevich et al., 2012). This system was applied to diverse and difficult sample types such as environmental biofilms (McLean et al., 2013a,b; Nurk et al., 2013a) which enabled the recovery of oral pathogens from a hospital sink biofilm (McLean et al., 2013b). This recent work represented only the third genome of the globally important pathogen *P. gingivalis* at the time (McLean et al., 2013b). The validation of this technique marks a new opportunity to capture pathogen genomes from environmental samples which may enable pathogen transmission between the environment and host to be better understood. Single cell genomic techniques are rapidly expanding the reference genomes available for oral and other body sites from the human microbiome through such high throughput platforms (Lasken, 2012). In particular many novel oral and gut bacterial genomes of varying finished quality will soon become publically available as part of the HMP effort. Many of these were chosen from the “100 most wanted” list of bacteria (Fodor et al., 2012) that reside in the human body but represent phylogenetic branches that do not have representative genomes. Recently, several uncultivated and difficult to cultivate oral bacteria such as members of the Tanerella genus [67], the Deltaproteobacteria [68] have now been sequenced with this approach. There are some 30 or more recognized candidate phyla still without a single representative cultivated member. Overall the impact of single cell genomics is immense as genomes from species that have so far eluded standard cultivation approaches are being captured. Not only are these providing interesting insights into novel metabolisms(McLean et al., 2013a; Rinke et al., 2013) but they are also being used as reference genomes to recruit DNA and RNA reads from other global sequencing studies(Rinke et al., 2013), ultimately providing a more comprehensive community level understanding of microbiomes.

## Linking function to phlyogeny

**Figure 4:**
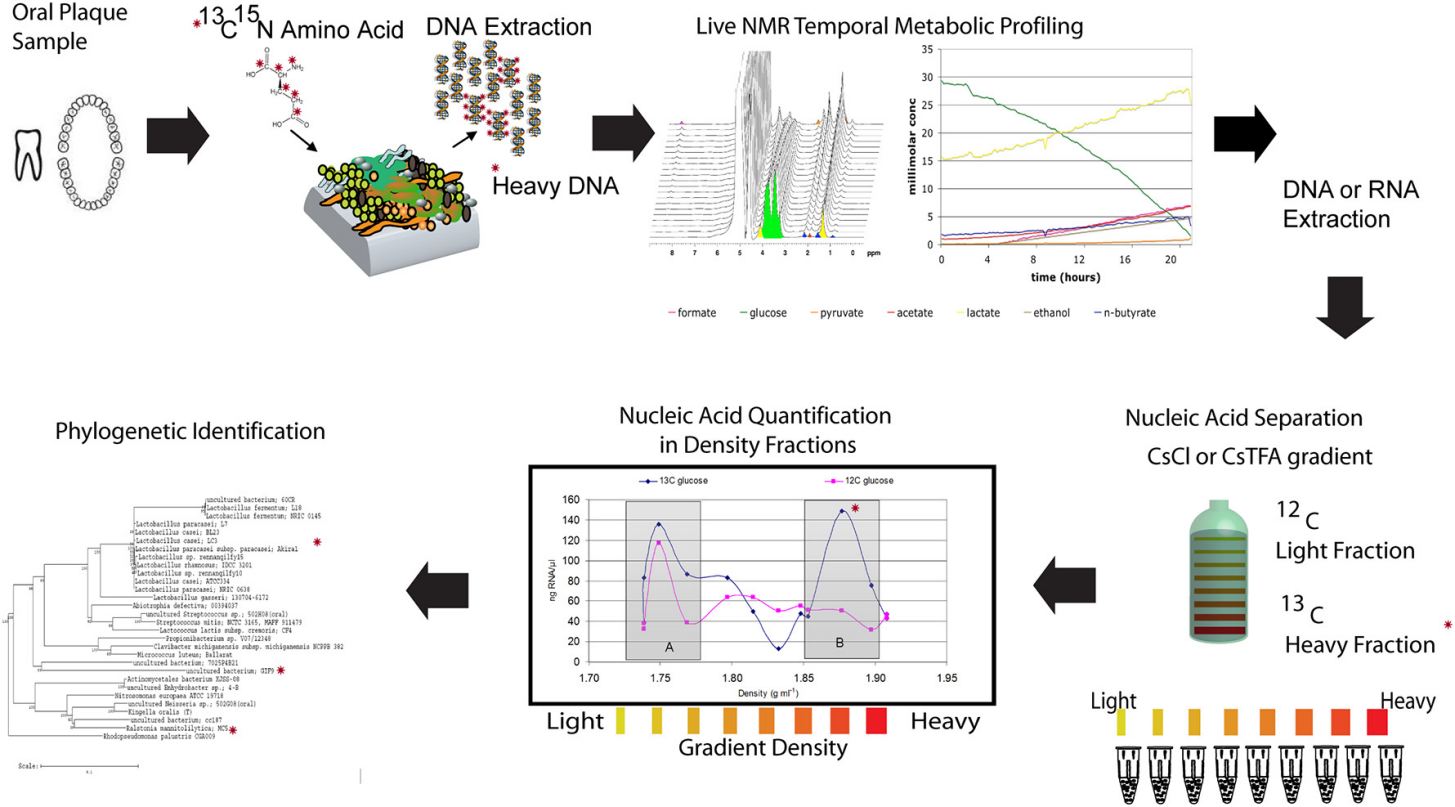
**Illustration of a NMR-SIP experimental procedure**. Combining nucleic acid based Stable Isotope Probing (SIP) with *in situ* non-invasive Magnetic Resonance Spectroscopy (MRS) to link real-time organic acid production measurements and the specific bacterial species active in oral biofilms. The experimental procedure involves incubating plaque samples from healthy juvenile human subjects with isotopically labeled carbon sources (^13^C-glucose or ^13^C-lactate) in a defined minimal medium under various pH and buffering conditions. The temporal metabolite profiles of these live samples can then be monitored by inserting the biofilms into the NMR and performing spectroscopy with ^1^H MRS followed by nucleic acid isolation and SIP to determine the active species (modified from McLean et al., 2012). The ^*^ indicates a heavy isotope labeled carbon or nitrogen that can be tracked in each panel.

As discussed earlier, determining which microbes are responsible for metabolizing substrates in a mixed microbial community is one of the biggest challenges in microbial ecology. SIP (Boschker et al., 1998; Radajewski et al., 2000) methods offer great potential to identify the cultivated and uncultivated microorganisms that metabolize and assimilate specific substrates in lab and field samples, and to identify metabolic networks that define functional microbial communities. Given what little is known about the metabolism of uncultivated bacteria and even metabolisms of known bacteria in the context of a diverse species background, the application of SIP to dental plaque holds considerable promise for meeting this challenge. Recent efforts in our laboratory have combined nucleic acid based SIP with the previously mentioned *in situ* non-invasive Magnetic Resonance Spectroscopy (MRS) to link real-time organic acid production measurements and the specific bacterial species active in oral biofilms (McLean et al., 2012) (Figure 4). The experimental procedure involved incubating plaque samples from healthy juvenile human subjects with isotopically labeled carbon sources (^13^C-glucose or ^13^C-lactate) in a defined minimal medium under various pH and buffering conditions. The temporal metabolite profiles of these live samples were monitored by inserting the biofilms into the NMR and performing spectroscopy with ^1^H MRS. The study was based on the working hypothesis that a low pH environment simulates the time at which the dissolution rate is highest and only those bacteria that can tolerate and continue to metabolize ^13^C-glucose (and byproducts) will be detected in the heavy labeled isotope fractions. Using this novel application, we demonstrated that this approach allows reconstruction the community interactions by identifying potential acid active species (including uncultivated species) under a set of conditions that were relevant to the enamel (hydroxyapatite) dissolution (McLean et al., 2012). For example, our initial findings through the use of SIP confirmed that species other than the model species of mutans streptococci are metabolizing at low pH. Specifically, Lactobacilli (which are known cariogenic species) are highly active at pH 5.5 and also pH 4.5 within intact plaque. Additionally through the use of ^13^C-labeled lactate, SIP gave some indication of the diversity of species able to metabolize lactate and byproducts. In the future, addressing these types of outstanding questions with the use of advanced methods to link phylogeny with function will enable assigning key functions to both known and uncultivated species thereby building the overall knowledge base.

## Advanced biofilm models: enabling connection between single and mixed species approaches

Most model systems for biofilms have utilized single-species systems, with the goal of understanding the various processes that occur during the “life cycle” of a biofilm formed in the laboratory. Model systems can drive technological advances since they provide a test bed for new technologies and approaches. They have proven to be valuable for the elucidation of the fundamental aspects of oral microbial biofilm formation. In general, growth models attempt to mimic *in situ* conditions as much as possible and to control input and environmental parameters so that cell-cell interactions can be understood. While some may question the relevance of this approach for understanding *in situ* biofilms, it is a necessary step between studying individual members and directly sampling and interpreting the highly complex, uncontrolled environment that the human oral microbiome represents.

There are considerable difficulties inherent in the development of a multi-species biofilm model system. A range of approaches and microbial communities of varying complexity has been utilized, with different uses, strengths, and limitations. Undoubtedly, each is a compromise between the actual microbiome conditions and the simplification and controllability necessary to gain meaningful, useful results in the laboratory. The model system thus tend to be limited in the range of uses, with potentially high variability, and difficult in terms of the interpretation of results (Sissons, 1997). The further development of existing model systems and the development of new complex multi-species systems that are particularly well suited to address fundamental questions of biofilm community structure and function are truly needed. With such systems, we can begin to address some of the issues specific to the study of biofilms, including: (1) temporal and spatial heterogeneity in environmental parameters; (2) spatial heterogeneity in growth rates; (3) small sample sizes; and importantly, (4) fast dynamic temporal changes in metabolites and gene expression.

**Figure 5:**
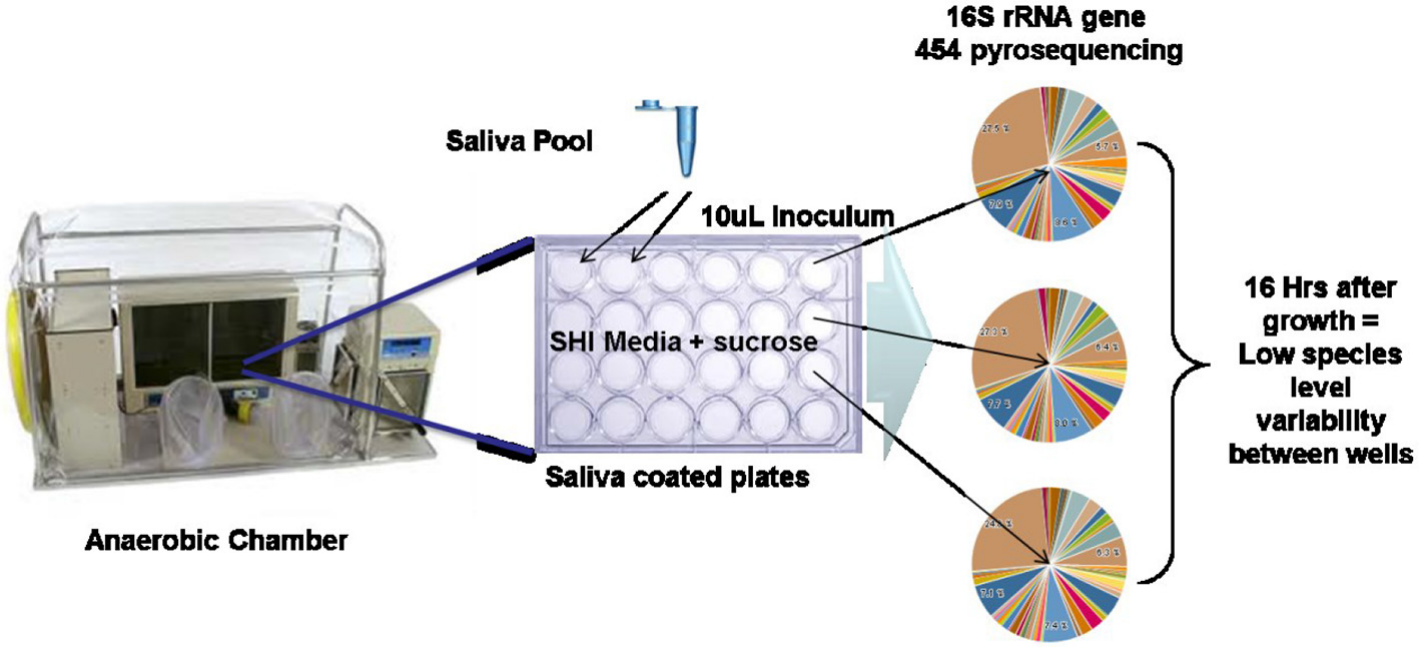
***In vitro* oral biofilm model system (Edlund et al., 2013)**. Schematic of *in vitro* model system growth and 16S rRNA gene sequencing results indicating high reproducibility of taxonomic abundance and carriage between wells.

The challenge of establishing oral biofilm models that attempt to approach the complexity of the species seen *in vivo* is an area that has seen significant progress. These range from using defined mixtures of 10 or more species (Bradshaw et al., 1989, 1994, 1996) as well as using plaque or saliva inoculum and various media formulations including real and simulated saliva (Palmer et al., 2006; Kolenbrander et al., 2010). Recently, efforts to develop a stable, highly diverse mixed microbial *in vitro* biofilm model of the oral cavity was achieved through iterative manipulation of media components and monitoring the species diversity compared to a pool of saliva from healthy subjects (Tian et al., 2010). Edlund et al. (2013) describe how this multi-species model system was developed and investigated. The general outline of the easily employed model is shown in Figure 5. We discovered that there was remarkable reproducibility of species occurrence and even abundance between biofilm in each well of a 24 well plate, between batches and even between laboratories with independent media batches. Although surprising, it explained the highly repeatable pH profiles after carbohydrate addition nearly identical to the Stephan pH profiles (Stephan, 1943) (Figure 1) seen from early studies in the 1940s and documented in nearly every *in vivo* and *in vitro* plaque sample to date. In light of the recent HMP studies which documented a high taxonomic variability between individuals but a highly similar functional genomic content (Consortium, 2012b), this stable physiology is likely a consequence of this encoded metabolism and is probably specific to the conditions in each body site. The use of a laboratory model system with functional and species reproducibility maintaining a highly complex bacterial diversity that also supports the growth of otherwise uncultivated species is desirable as it can be manipulated and studied over a longer period of time in a controlled environment. A validated system that fulfills these criteria and that can be readily used for example to target changes in taxa, regulation of metabolic pathways and signaling molecules by using next generation sequencing (NGS) and “omics” methodologies (SIP, metatranscriptomics). Specifically such a model system will help facilitate experimental approaches that seek answers to questions related to the roles each bacteria plays in the overall structure and function of the human oral microbiome.

## Capturing gene expression of entire communities: metatranscriptomics

NGS technologies have provided a new way to assess the gene expression (transcription activity) of bacteria, predominately referred to as RNA-seq (Mader et al., 2011; Pinto et al., 2011). The strength of this methodology relies on the very large number of sequence reads generated with NGS platforms. This enormous quantity of reads generated; now allow the number of expressed transcripts (mRNA) to be determined by mapping reads to reference genomes or assembled *de novo*. Uniquely, RNA-seq also permits the quantification of novel transcripts such as small RNAs within intergenic regions that might not have been previously predicted and targeted by microarrays or qRT-PCR primers. Furthermore, the decreasing costs associated with RNASeq in comparison to conventional DNA-microarray hybridization techniques, justifies the use of this approach. Until recently however, techniques used to characterize gene expression in more complex natural microbial communities (Shi et al., 2009) have been challenged by the overwhelming genetic diversity and metabolic complexity of these consortia (Shi et al., 2009; Moran et al., 2013). The working hypothesis for metatranscriptomics applied to microbial communities is that transcripts associated with the active genes responding to each stage of the described interactions will be more highly abundant. Specifically, metatranscriptomic analyses can be applied to communities in a number of defined interactions to delineate the expressed genes thereby moving closer to the real functions of the bacteria under specific conditions. Species-specific genes in oral bacteria that are up-regulated in response to specific conditions such as low pH for example would ideally give insight into the mechanisms that bacteria use for acid tolerance and maintenance of cytoplasmic pH. Specifically tagging and tracking these genes or the cell response within a species is then possible, as recently shown for tracking extracellular pH with *S. mutans* (Guo et al., 2013).

## General analysis of genomic and metatranscriptomic datasets

Although many groups approach the collection and analyses of global mRNA datasets, the generalized workflow for metatranscriptomics involves mapping reads onto reference and/or assembled genomes/metagenomes using such short read mapping tools. The counts for each genomic region can then be extracted and tabulated. Comparative gene expression analyses between sequencing libraries can be performed using tools and approaches developed to handle the dynamic range of RNA-seq datasets as opposed to methods developed for microarrays. Importantly, to determine the significance of the genes expressed, approaches for normalization of the data built into these processing tools and the statistical tests used within are being developed and tested. Gene transcription boundaries, regulatory regions and expression of small RNA can be analyzed in detail by using more sophisticated approaches once they are identified in the genome of interest. In addition, *de novo* assembly of the transcripts can then be performed which allows coding regions to be determined from the community that could assemble into new and novel genes. These open reading frames (ORFs) can be annotated and compared to existing genomes representing closely related bacterial genomes. Analysis of the metatranscriptomic data ideally allow identification of both structural and regulatory genes coding for the molecular mechanisms involved in bacterial physiology.

In general, the types of functional analyses approaches looking at global metatranscriptome data include:

1. **Comparative metatranscriptomics**. Differential expression (DE) of genes or orthologous clusters of genes in regards to a reference condition or temporal change in expression patterns.
2. **Functional Analyses**. Annotation information such as enzyme commission (EC) number assignments, COGs and hidden Markov models (HMMs) can be assigned to reads that assembled into partial or full genes and used to construct metabolic pathways to the extent possible to gain new insights into metabolic pathways present in a community. EC number assignments, for example, can be used to populate KEGG metabolic maps and can be enhanced with expression level information. The assignment of EC numbers, COGs, and HMMs can then be used to perform statistical analyses to determine over-representation of pathways/processes in the transcriptomes from each sample relative to one another.
3. **Phylogenetic profiling**. Patterns of up and down-regulated gene families or proteins in different genomes can be compared. Organisms sharing a particular expression pattern for example can be considered functionally similar thus indicating possible synergistic or competitive interactions.

The transcriptome of oral bacterial communities are being explored both in terms of the potential differences in expression between health and disease in caries (Peterson et al., 2013; Benitez-Paez et al., 2014), periodontal disease (Duran-Pinedo et al., 2014; Jorth et al., 2014) and defined mixed communities containing sequenced oral bacteria (Frias-Lopez and Duran-Pinedo, 2012). There are many technical and bioinformatics based challenges still associated with interpretation of microbial gene expression patterns in mixed species biofilm communities. One of the major concerns that needs to be addressed is the existence of phylogenetically closely related strains in the community: the identification of a unique read to a given strain can accomplished only when a reference genome of that strain is available. For the present, in most microbiome communities, a great majority of reads do not map to any known reference genome or may be overlapping with a closely related genome. Using large databases of reference genomes enable read counts across multiple genomes to be divided to attempt to account for highly conserved genes present in multiple genomes.

Another large confounding parameter is the change in a given genome abundance between two samples that can skew differential gene expression when comparing samples sets such as between healthy vs. disease within *in vivo* collected samples. For example, if particular species abundance increases the number mRNA copies will increase and thus appear to be a differentially expressed gene between the sample sets. This is somewhat more tractable when studying controlled model systems that may not suffer from the large intra- and inter-personal taxonomic variance that can occur in samples collected directly from human subjects. Efforts to correct for this abundance change are not a straight forward task and likely will not be routinely implemented until further development, testing and validation. Given the discrepancy between 16S rRNA gene abundance and abundance measures using read mapping to a particular genome strain (using marker genes), the act of building out the reference genome with single cell genomics and standard cultivation approaches will greatly aid this effort. Future research including more fundamental approaches to validate metatranscriptomic data will prove the utility of this technique ultimately providing useful transcript biomarkers of health and disease.

## Summary and future directions

Much of what is known about all of the cultivated and sequenced oral bacterial species to date has been derived from pure culture approaches and laboratory experimentation, which likely does not reflect their actual behavior in complex microbial communities. Furthermore, as mentioned, roughly half of species identified through culture independent methods in the oral cavity are still only classified as uncultivated phylotypes. Many of these species have been found in deep cavities and therefore possibly linked to demineralization processes however their contribution to diseased states has not yet been established. Even low abundance members of a microbial community cannot be dismissed as inconsequential and in fact may express key properties that upset the balance and shift the metabolism of the community which for example, is the current belief with *Porphyromonas gingivalis* (Darveau et al., 2012; Hajishengallis et al., 2012). With the high level of effort to characterize the diversity and metabolic capacity encoded in the genomes of isolated bacteria associated with the oral cavity, a logical (and very important) next step is to increase our limited understanding of the process through community level physiological and molecular based studies. Such insights will help find new solutions to modulate the activity of the communities and steer them toward a healthy state. The huge challenges remaining, such as the vast uncultivated species and the lack of reference genomes currently limit this understanding. Capturing genomes of yet-to-be cultivated species will serve not only to gain insight into their potential physiology but will enable verification of this predicted metabolism using sequencing based approaches such as metatranscriptomics to measure the expression of genes while they are within the community. Methods such as single cell genomics to capture genomes from biofilms as well as innovative cultivation strategies such as the stable domestication from human communities to *in vitro* communities are key. Ultimately, once more genomes become available, we can apply concomitantly, the arsenal of approaches as described earlier including non-invasive imaging and metabolic analysis methods followed downstream by such tools as SIP and the expression profiling of all species. Notably, the involvement of virus component and the host responses add to the already enormous challenges. Logically, these techniques are best applied in more controlled and reproducible mixed species model systems first to gain baseline information and build solid databases of information. As the knowledge, technology and capabilities evolve, these can be more confidently applied to *in vivo* samples.

In future oral microbiome studies developing from these advancements, a particular emphasis can be placed on the discovery of low pH metabolism, low pH adaptations and organic acid production most relevant to mineral (hydroxyapatite) dissolution process as well as the metabolisms/species that are linked to healthy pH recovery phase. Overall the combination of these approaches on oral microbial systems of interest will reveal species (cultured and uncultured) involved in disease related processes and provide new insights into specific species, genes/domains, gene products, and metabolic pathways that define the synergistic and competitive contributions to both health and disease.

### Conflict of interest statement

The author declares that the research was conducted in the absence of any commercial or financial relationships that could be construed as a potential conflict of interest.
